# Spatiotemporal estimation of nutrient data from the northwest pacific and east asian seas

**DOI:** 10.1038/s41597-023-02602-4

**Published:** 2023-10-14

**Authors:** Gi Seop Lee, Jung Ho Lee, Hong Yeon Cho

**Affiliations:** 1 Marine Bigdata AI Center, Korea Institute of Ocean Science and Technology, Busan, 49111 South Korea; 2 Data Tech Team, SK Telecom, Seoul, 02598 South Korea

**Keywords:** Marine chemistry, Statistics, Scientific data, Marine chemistry

## Abstract

Nutrient data obtained from field observations have the potential to enhance our understanding of oceanic biogeochemical cycling and productivity changes. In particular, long-term nutrient data can provide valuable information on the links between climate change and biogeochemical changes. However, unlike other observational variables such as sea surface temperature, nutrient data are limited in terms of their broad-scale observations and automated sensor-based measurements. In this study, we analyzed nitrate and phosphate data obtained from coastal regions in Northeast Asia and the northwest Pacific from 1980 to 2019 using the spatiotemporal kriging technique and provide results in a spatiotemporal grid format. The data are available at monthly intervals and may be attractive to researchers in the fields of oceanography, marine ecology, and marine biogeochemistry at the climate change scale. Furthermore, sharing the source code of the data production process can contribute to better long-term data reproduction in the future.

## Background & Summary

The northwest Pacific and adjacent eastern Asian waters are known for their high primary productivity^1–3^, which has been examined in various marine chemical and biological studies, including those on water quality changes and material cycling. Nutrient data play a crucial role in these studies, with nitrogen and phosphorus being especially significant, as they influence the growth and reproduction of phytoplankton and shape the area’s phytoplankton species composition^4–9^. Human activities, such as artificial nitrogen input in densely populated regions such as eastern China and western Europe, affect the ocean’s biogeochemical structure^10–13^.

Coastal waters near East Asia, including the heavily impacted Yellow Sea and East China Sea, the unique East Sea with relatively lower human impact, and the northwest Pacific influenced by the Kuroshio current, are influenced by both natural and human factors, leading to complex changes in nutrient levels^14^. Nutrient supply from the deep sea has been reported to have decreased worldwide due to the strengthening of the stratification^1–3,15^, while artificial supply through the atmosphere and rivers has increased in the East Asian waters^11,12,16–18^. Thus, understanding the long-term changes in nutrient levels in this region is crucial, and various perspectives are being studied to comprehend the phenomenon and predict future changes^6,19–21^.

From the perspective of the spatial estimation of ocean information, studies providing gridded data such as OISST (Optimum Interpolation SST [Sea Surface Temperature]) are ongoing^22–24^. In addition, research is being conducted to remove the bias of numerical models using spatial estimation techniques such as Kriging^25^. However, these techniques mainly focus on temperature and salinity. Nutrient data, however, primarily rely on *in situ* observations, as satellite remote sensing and unmanned equipment cannot cover them. Efforts have been made to provide monthly gridded nutrient data for the North Pacific region^26–28^, but 4D gridded nutrient data (*x, y, z, t*) that consider both spatial and temporal variations are limited to some reanalysis data using numerical models^29^.

This study uses 40 years of nutrient concentration observations from 1980 to 2019 to spatially and temporally optimize the data for the northwest Pacific Ocean (N25–45°, E121–145°) into a gridded format. The estimated nutrient grid data were validated through a verification process and are presented (with validation errors shown) in the modeled results.

## Methods

**Fig. 1:**
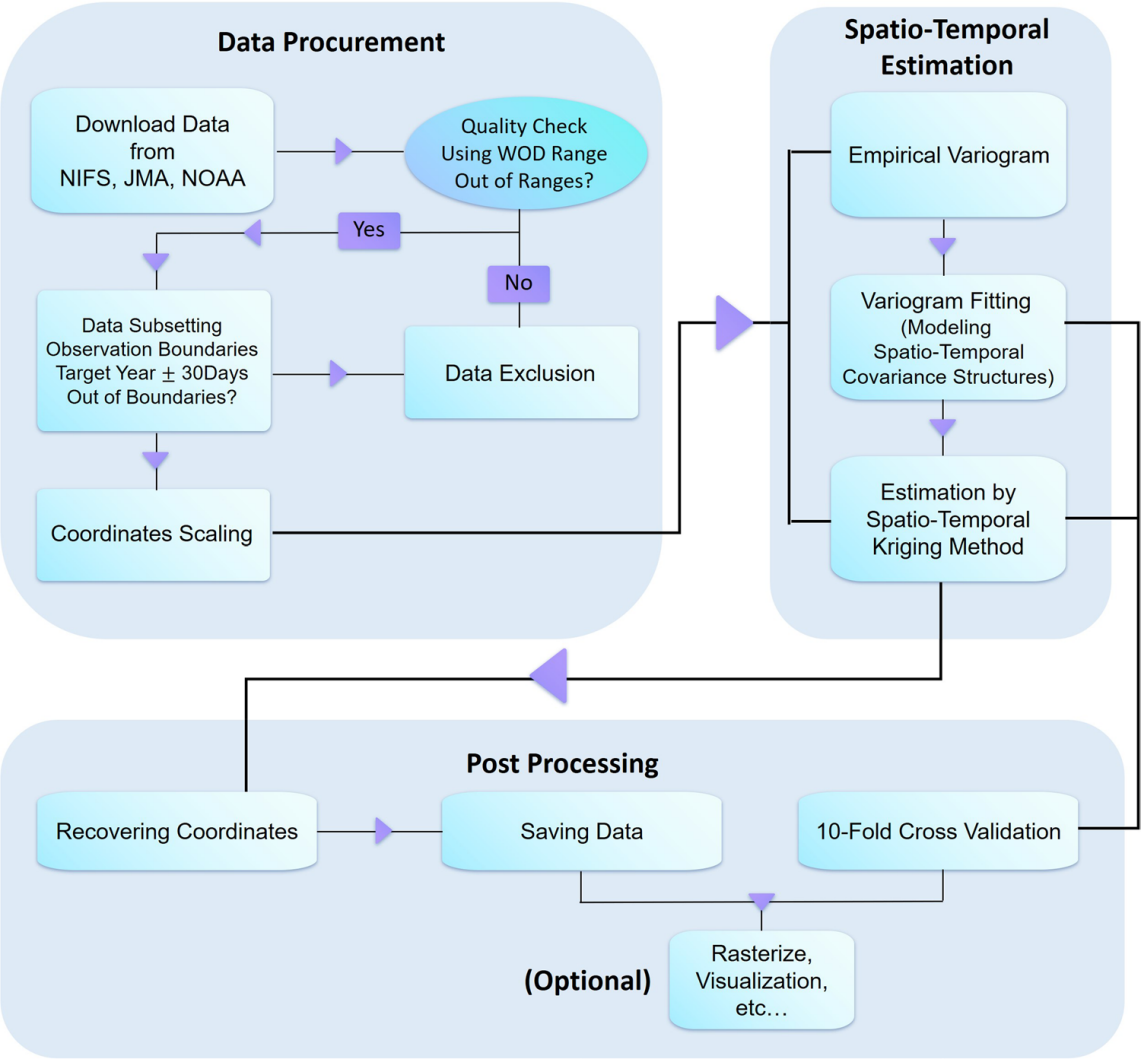
Workflow of the study. Nutrient and depth data were downloaded from various sources. The nutrient data obtained from different sources were compiled into a common data format, and data within the valid range of time, space, and nutrient variables were selected. Then, the refined data were used for variogram modeling and spatiotemporal kriging. 10-fold cross-validation was performed to indicate estimation errors, and the data were saved.

The procedure used to create gridded data is summarized in Fig. 1. This section outlines the steps involved in transforming raw observational data into nutrient grid data and validating the results. All procedures were executed using the R programming language (R core team, 2023)^30^. The data production process consisted mainly of three steps: data collection and preprocessing, spatiotemporal estimation, and postprocessing and validation. These steps will be discussed in further detail below.

### Data procurement

The data analyzed in this study were acquired from the National Institute of Fisheries Science (NIFS) Serial Oceanographic Observation (SOO, https://www.nifs.go.kr/kodc/eng/eng_coo_list.kodc)^31^, Japan Meteorological Agency (JMA) Oceanographic and Marine Meteorological Observations by Research Vessels (OMMORV, https://www.data.jma.go.jp/gmd/kaiyou/db/vessel_obs/data-report/html/ship/ship_e.php)^32^, and topographic data from the National Oceanic and Atmospheric Administration (NOAA) Earth TOPOgraphy(https://www.ncei.noaa.gov/maps/grid-extract/)^33,34^. The data specifications, including file format, observational period, spatiotemporal resolution, and accessible URLs, are presented in Table 1.

**Table 1:** Data specifications for SOO, OMMORV, and ETOPO 2022.

Title	Provider	Format	Temporal Range	Resolution	Access URL
Serial Oceanographic Observation (SOO)	NIFS, Korea	.txt,.xls(excel),.csv(in korean)	1967–2022 1980–2022 (used)	Bimonthly (temporal)	https://www.nifs.go.kr/kodc/eng/eng_coo_list.kodc (in English) https://www.nifs.go.kr/kodc/soo_list.kodc (in Korean)
Oceanographic and Marine Meteorological Observations by Research Vessels, Hydrographic data (OMMORV)	JMA, Japan	.E (fixed width)	1964–2022 1980–2022 (used)	Seasonally (temporal)	https://www.data.jma.go.jp/gmd/kaiyou/db/vessel_obs/data-report/html/ship/ship_e.php (in English, >1997) https://warp.ndl.go.jp/info:ndljp/pid/11160873/www.data.jma.go.jp/gmd/kaiyou/db/vessel_obs/data-report/html/ship/efile_NoS2_e.html (in English, <2010)
ETOPO 2022	NOAA, US	.tif	—	15 arc sec (spatial)	https://www.ncei.noaa.gov/maps/grid-extract/

The table includes information on data providers, file formats, observational periods, spatiotemporal resolutions, and accessible URLs.

The OMMORV data are currently accessible for download starting from 1997, with earlier data available in the JMA Data Report of Oceanographic Observations Special Issue^35^. To avoid duplications, any potential overlap in the data were excluded from the spatiotemporal estimation process.

### Nutrient data

The **SOO data** can be obtained by specifying the region, line, station, observation date, and depth. These data comprise ten water quality parameters, including temperature, salinity, dissolved oxygen concentration, nitrate concentration, and phosphate concentration. The data have been collected since 1961 with a bimonthly (February, April, June, August, October, and December) measurement frequency. Although the station and line locations may have undergone slight changes, as of 2020, data from 207 stations along 25 lines have been accumulated.

The **OMMORV data** can be obtained through the provided link associated with each research vessel. The hydrographic data, saved with an ‘.E’ extension, were used in this study. The format of the data underwent a change in 2010 and is now classified into two versions, ‘E2.x’ and ‘E3.x’. The data are a 126-byte ASCII record in a fixed width format, with observation information and items arranged at regular intervals. Detailed information on the format of the data is available separately^36^.

To ensure compatibility, the nutrient data from both SOO and OMMORV underwent unit conversion. The original units of μmol/kg were changed to μmol/L, and the density was calculated based on the recorded water temperature and salinity data. This calculation was carried out assuming standard atmospheric pressure (10.1325 dbar) with the *gsw* library in the R programming language^37,38^. The SOO and OMMORV data were merged into a table of 2,023,251 observations and 16 variables, and the missing information for each variable is shown in Table 2.

**Table 2:** The basic description of the dataset merged from SOO and OMMORV.

Variables	Description	Unit	class	N of missing
*Nat*	Nation		Character	—
*st*	Station		Character	—
*date_time*	Date and Time		POSIXct	4,150
*lat*	Latitude	Degree(°)	Numeric	—
*long*	Longitude		Numeric	—
*tm_x*	Coordinate x, TM projected long	m	Numeric	—
*tm_y*	Coordinate y, TM projected lat		Numeric	—
*depth*	Depth		Numeric	766,492
*wt*	Water Temperature	°C	Numeric	632,880
*sal*	Salinity	psu	Numeric	633,813
*pH*	potential of Hydrogen	—	Numeric	1,611,517
*DO*	Dissolved Oxygen	ml/L (NIFS) μmol/kg (JMA)	Numeric	803,932
*PO4*	Phosphate	μmol/L (NIFS, JMA <2010 winter) μmol/kg (JMA ≥ 2010 spring)	Numeric	1,160,474
*NO2*	Nitrite		Numeric	1,291,329
*NO3*	Nitrate		Numeric	1,296,471
*SiO4*	Silicate		Numeric	1,619,079

The preprocessed R object name and its description, unit, data type, and number of missing data are specified.

The bathymetry used in this study utilized NOAA’s ETOPO 2022 data, which is the second release of these data following ETOPO1^33,39^. The water depth data can be obtained either by downloading the desired area data from the following URL or by using the R Package *marmap*^40^. In this study, the water depth data within the range of N25–45° and E121–145° were processed at 10-minute intervals using the *getNOAA.bathy* function from the *marmap* library. The computation was performed on grid points that were densely set from the surface to the 9,000 m depth range using the standard depth from WOD18^41^. However, to reduce the computational load, the number of depth grids was reduced from 137 to 43. Depth intervals were created at 20 m intervals for the 0–200 m range, 100 m intervals for the 200–2000 m range, and 500 m intervals for the 2000–9000 m range to produce the grids.

### Spatiotemporal estimation

The spatiotemporal kriging (STK) approach was used to transform the irregular nutrient data collected from the SOO and OMMORV sources into spatiotemporal grid data. This method has been widely used in various fields^42–45^ and distinguishes itself from those in previous studies by considering the vertical dimension in the 4-dimensional spatiotemporal estimation. The R libraries *gstat* and *spacetime* were utilized for maximum 3-dimensional spatiotemporal estimation^46–48^; however, a custom function had to be developed, as 4-dimensional coordinates are not supported by these libraries.

Kriging with External Drift (KED) was applied for nutrient estimation in unmeasured spatiotemporal areas using spatiotemporal coordinates as the auxiliary variables. KED is also referred to as universal kriging when the drift is limited to spatial coordinates^49,50^. The unmeasured point’s estimation at a specific time point is represented as a weighted combination of the spatial trend and the residual from the regression model at the measured point as in Eq. (1).

1 $$\widehat{z}\left({x}_{0}\right)={\sum }_{k=1}^{m}{\omega }_{k}{f}_{k}\left({x}_{0}\right)+{\omega }_{0}+{\sum }_{i=1}^{n}{{\rm{\lambda }}}_{i}e\left(z\left({x}_{i}\right)-\bar{z}\right)$$

where *x*_*i*_ and *x*_0_ represent the coordinates of the observation and the target location, respectively, and the 4-dimensional coordinate structure including horizontal, vertical, and temporal dimensions is represented as $\left[x,y,z,t\right]=\left[{x}_{1},\cdots \,,{x}_{m}\right]$. The subscript *i* denotes the observed location, and 0 denotes the location of interest for prediction. *f*_*k*_ (*x*_0_) is a function representing the average spatiotemporal variation, and a linear function is used. *ω*_*k*_ is the coefficient of the regression function *f*_*k*_ (*x*_0_), and *ω*_0_ is the Lagrange parameter to remove bias. *e* is the residual of *f*_*k*_ (*x*_0_), and *λ*_*i*_ represents the weight coefficient of *e*.

The optimal coefficients (*ω*_*k*_, *ω*_0_, *λ*_*i*_) that satisfy the condition of minimizing the error variance in Eq. (2) are derived in the form of Eq. (3), and it is solved as shown in Eq. (4).

2 $$\min {\left[\widehat{z}\left({x}_{0}\right)-z\left({x}_{0}\right)\right]}^{2}$$

The process of finding the solution in the form of a matrix equation is shown in Eqs. (3, 4), and the block matrices that make up the overall matrix equation are constituted as in Eq. (5–11). The bold symbols indicate the block matrix.

3 $${{\boldsymbol{C}}}^{{\boldsymbol{KED}}}\cdot {{\boldsymbol{\lambda }}}^{{\boldsymbol{KED}}}={{\boldsymbol{C}}}_{{\bf{0}}}^{{\boldsymbol{KED}}}$$

4 $${\lambda }^{{\boldsymbol{KED}}}={{\boldsymbol{C}}}^{{{\boldsymbol{KED}}}^{-1}}\cdot {{\boldsymbol{C}}}_{{\bf{0}}}^{{\boldsymbol{KED}}}$$

5 $${{\boldsymbol{\lambda }}}^{{\boldsymbol{KED}}}=\left[\begin{array}{c}{{\boldsymbol{\lambda }}}_{{\boldsymbol{i}}}\\ {\omega }_{0}\\ {{\boldsymbol{\omega }}}_{{\boldsymbol{k}}}\end{array}\right],i=1,\ldots ,n;k=1,\ldots ,m$$

6 $${{\boldsymbol{C}}}^{{\boldsymbol{KED}}}=\left[\begin{array}{ccc}{{\boldsymbol{\sigma }}}_{{\boldsymbol{ij}}}^{2} & {{\boldsymbol{I}}}_{{\boldsymbol{n}}} & {\boldsymbol{X}}\\ {{\boldsymbol{I}}}_{{\boldsymbol{n}}}^{{\boldsymbol{T}}} & {\bf{0}} & {{\bf{0}}}_{{\boldsymbol{m}}}\\ {{\boldsymbol{X}}}^{{\boldsymbol{T}}} & {{\bf{0}}}_{{\boldsymbol{m}}}^{{\boldsymbol{T}}} & {\bf{0}}\end{array}\right]$$

7 $${{\boldsymbol{C}}}_{{\bf{0}}}^{{\boldsymbol{KED}}}=\left[\begin{array}{c}{{\boldsymbol{\sigma }}}_{{\bf{0}}{\boldsymbol{j}}}^{2}\\ 1\\ {{\boldsymbol{X}}}_{{\bf{0}}}\end{array}\right]$$

where ***X*** is the observed coordinates, ***X***_**0**_ is the target coordinates, and ~ represents the min-max scaled coordinates. ***I***_***n***_ is a unit vector of *n* × 1, **0**_***m***_ is a zero vector of 1 × *m*, and **0** is a zero matrix of *m* × *m*. The superscript T on a matrix represents the transpose.

8 9 $${\boldsymbol{X}}=\left[\begin{array}{ccc}{({\widetilde{x}}_{1})}_{1} & \cdots  & {({\widetilde{x}}_{m})}_{1}\\ \vdots  & \ddots  & \vdots \\ {({\widetilde{x}}_{1})}_{n} & \cdots  & {({\widetilde{x}}_{m})}_{n}\end{array}\right]=\left[\begin{array}{cccc}{\widetilde{x}}_{1} & {\widetilde{y}}_{1} & {\widetilde{z}}_{1} & {\widetilde{t}}_{1}\\ \vdots  & \vdots  & \vdots  & \vdots \\ {\widetilde{x}}_{n} & {\widetilde{y}}_{n} & {\widetilde{z}}_{n} & {\widetilde{t}}_{n}\end{array}\right],=\left[\begin{array}{c}{\widetilde{x}}_{0}\\ {\widetilde{y}}_{0}\\ {\widetilde{z}}_{0}\\ {\widetilde{t}}_{0}\end{array}\right]$$

10 11 $${{\boldsymbol{\sigma }}}_{{\boldsymbol{ij}}}^{{\bf{2}}}={{\boldsymbol{\gamma }}}_{{\boldsymbol{st}}}^{{\boldsymbol{SM}}}({{\boldsymbol{h}}}_{{\boldsymbol{ij}}},{{\boldsymbol{u}}}_{{\boldsymbol{ij}}}),{{\boldsymbol{\sigma }}}_{{\bf{0}}{\boldsymbol{j}}}^{2}={{\boldsymbol{\gamma }}}_{{\boldsymbol{st}}}^{{\boldsymbol{SM}}}({{\boldsymbol{h}}}_{{\bf{0}}{\boldsymbol{i}}},{{\boldsymbol{u}}}_{{\bf{0}}{\boldsymbol{i}}})$$

where the matrices ${{\boldsymbol{\sigma }}}_{{\boldsymbol{ij}}}^{{\bf{2}}}$ and ${{\boldsymbol{\sigma }}}_{{\bf{0}}{\boldsymbol{j}}}^{{\bf{2}}}$ are calculated based on the spatiotemporal variogram ${\gamma }_{st}^{SM}\left(h,u\right)$ estimated from the observational data. The variogram represents the change in covariance with respect to distance and time, reflecting the strength of the correlation between data points as a function of spatial and temporal distance. ${\gamma }_{st}^{SM}\left(h,u\right)$ is expressed as a function of the spatial distance *h* and the temporal distance *u* (12, 13).

12.1 $${h}_{ij}=\sqrt{{\left({\widetilde{x}}_{i}-{\widetilde{x}}_{j}\right)}^{2}+{\left({\widetilde{y}}_{i}-{\widetilde{y}}_{j}\right)}^{2}+{\left({\widetilde{z}}_{i}-{\widetilde{z}}_{j}\right)}^{2}}$$

12.2 $${h}_{0i}=\sqrt{{\left({\widetilde{x}}_{0}-{\widetilde{x}}_{i}\right)}^{2}+{\left({\widetilde{y}}_{0}-{\widetilde{y}}_{i}\right)}^{2}+{\left({\widetilde{z}}_{0}-{\widetilde{z}}_{i}\right)}^{2}}$$

13.1 $${u}_{ij}=\sqrt{{\left({\widetilde{t}}_{i}-{\widetilde{t}}_{j}\right)}^{2}}$$

13.2 $${u}_{0i}=\sqrt{{\left({\widetilde{t}}_{0}-{\widetilde{t}}_{i}\right)}^{2}}$$

The ${\gamma }_{st}^{SM}\left(h,u\right)$ was fitted using the Sum-Metric (14) model^51,52^. The Spherical model (15) was applied equally to the *γ*_*s*_, *γ*_*t*_, and *γ*_*joint*_ models.

14 $${\gamma }_{st}^{SM}\left(h,u\right)={\gamma }_{s}\left({h}_{ij}\right)+{\gamma }_{t}\left({u}_{ij}\right)+{\gamma }_{joint}\left(\sqrt{{h}_{ij}^{2}+{\left(\kappa {u}_{ij}\right)}^{2}}\right)$$

15 $$\gamma \left(h\right)=Var\left(z\right)-{C}_{0}\left(1.5\frac{h}{a}-0.5{\left(\frac{h}{a}\right)}^{3}\right)+b$$

**Fig. 2:**
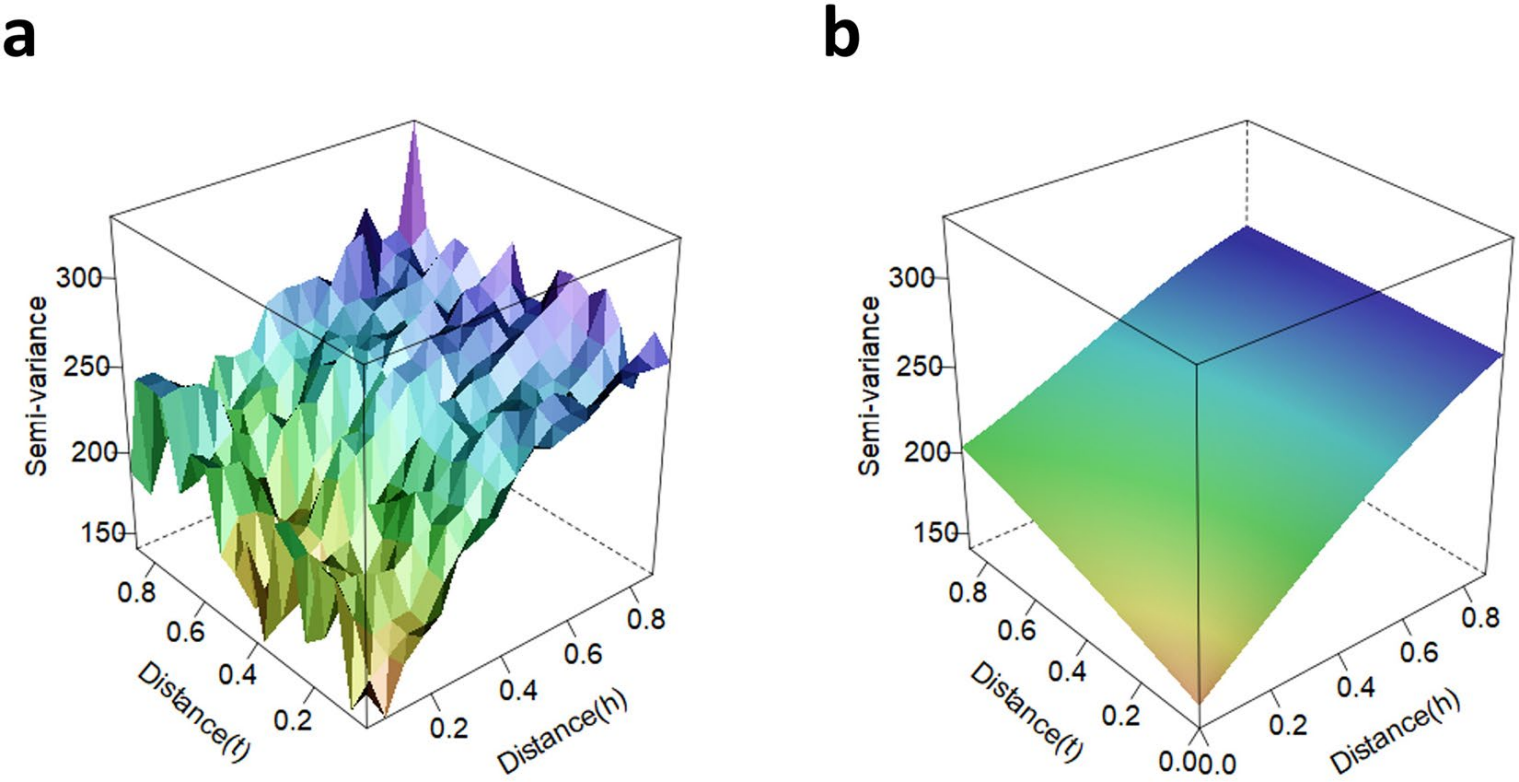
Example of empirical (**a**) and fitted (**b**) variograms. The nitrate data from 2013 were used and fitted using the Sum-Metric model. The spatial and temporal distances were min-max scaled. The semivariance of the z-axis represents the inverse correlation with respect to spatiotemporal distance.

In the variogram model, *h* is the separation distance (generally, spatial distance), *a* is the range, and *b* is the nugget. The minimum of the observational data is *C*_0_ + *b*, and *κ* is an anisotropic parameter for time. Each parameter was optimally estimated using the L-BFGS-B algorithm^46,53^. An example of spatiotemporal variogram modeling using observed nitrate data from 2013 is provided in Fig. 2.

When computing the actual $\widehat{z}\left({x}_{0}\right)$, only the estimated *λ*_*i*_ is used, and the regression coefficient *ω*_*k*_, which determines the spatial average variation, is calculated using Eq. (16) as a constraint.

16 $$\widehat{z}\left({x}_{0}\right)={\sum }_{i=1}^{n}{\lambda }_{i}{z}_{i}$$

The prediction results of Eq. (16) are accompanied by the indicator of estimation uncertainty, the error variance, as provided in Eq. (17).

17 $${\sigma }_{KED}^{2}={\sigma }^{2}-{\sum }_{i=1}^{n}{\lambda }_{i}{\sigma }_{0i}^{2}+{\sum }_{k=0}^{m}{\omega }_{k}{f}_{k}\left({x}_{0}\right)$$

## Data Records

The reproduced data are provided in comma-separated values (.csv) and R Data (.RData) file formats, with processing codes written in the R language^54^. The code for reading, analyzing, and visualizing the data can also be used to update the data. The data provided in CSV format consist of spatiotemporal coordinates (x, y, z, t), estimated values of nitrate or phosphate, and error variances of kriging. The error variances provide quantitative information on the magnitude of estimation errors and can be utilized in future conditional simulations. R Data (.RData) is a binary data format that can be directly loaded into memory using the *load* function in the R programming language for immediate use.

The dataset is projected in the Lambert azimuthal equal-area projection method with the following coordinate reference system (CRS):

“+*proj=laea* +*lat_0* = *34.53333* +*lon_0* = *137.0698* +*x_0* = *0* +*y_0* = *0 +datum* = *WGS84* +*units* = *m* +*no_defs* +*ellps* = *WGS84* +*towgs84* = *0,0,0*”

The data can be converted back to the longitude and latitude coordinate system using the following CRS:

“+*proj* = *longlat* +*datum* = *WGS84* +*no_defs* +*ellps* = *WGS84* +*towgs84* = *0,0,0*”

Coordinate transformation using R can be performed using spatial data libraries such as *sp* and *sf*’^55–57^.

However, the conversion process may introduce slight errors, resulting in longitude and latitude coordinates with nonuniform degree intervals. Therefore, interpolation methods such as aggregation, nearest neighbor, or bilinear interpolation may be necessary for the stretched grid.

## Technical Validation

**Fig. 3:**
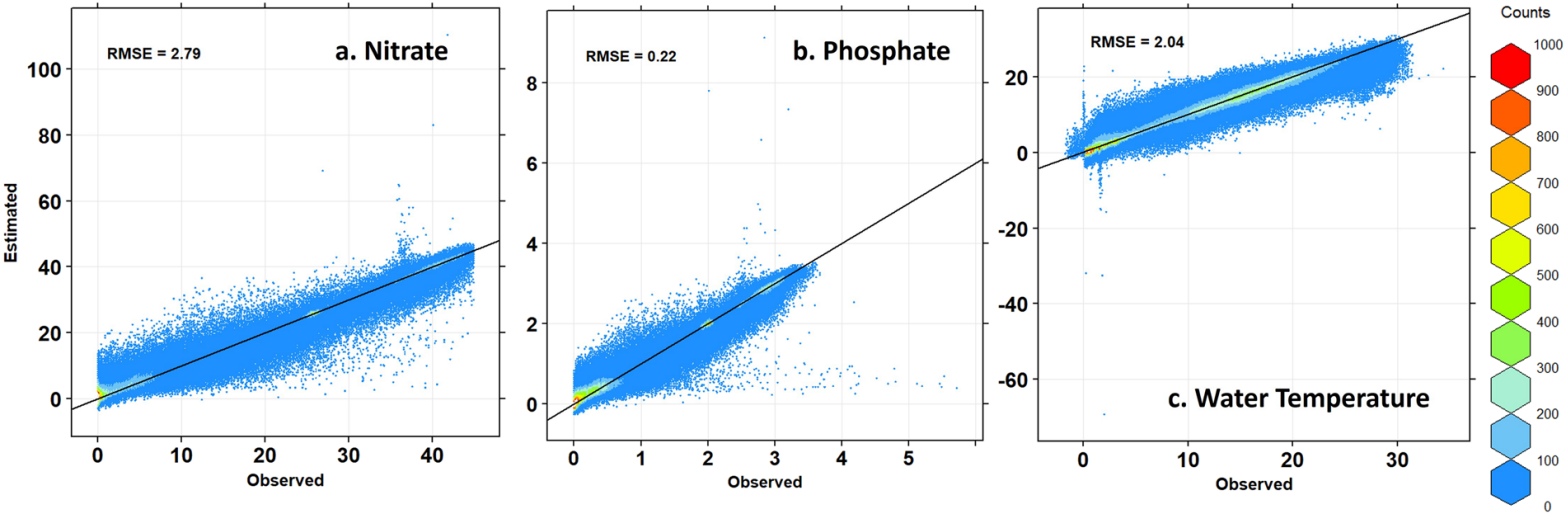
10-fold cross-validation results using observational data from 1980 to 2019, spanning a total of 40 years(**a**: nitrate, **b**: phosphate, **c**: water temperature). Some values fall outside of the valid range, but many data points are densely distributed around the 1:1 line (black solid line). Due to the nature of KED, concentration values calculated as negative values can be converted to 0 or detection limit values.

The performance of the estimation model was evaluated using 10-fold cross-validation for spatial estimation results obtained through STK (Fig. 3, Table 3). Note that Simple and Ordinary Kriging always predict values that are less than or equal to the maximum observed value, while KED can predict values that are greater or smaller than the neighboring observed values. Therefore, if an estimated value falls outside the range of the WOD18 standard, it may need to be adjusted to a value within the range before interpretation. In this case, negative concentration values were replaced with 0. The root mean square error (RMSE), mean absolute error (MAE), and adjusted coefficient of determination $\left({R}_{adj}^{2}\right)$ were used as performance evaluation metrics (18)^58^

18 $${R}_{adj}^{2}=\frac{n-1}{\left(n-m-1\right)\left(1-{R}^{2}\right)}$$

Where *n* is the number of data points and *m*(=4) is the number of predictor variables used in the estimation.

**Table 3:** The error evaluation metrics for the 10-fold cross validation, including the root mean square error (RMSE), mean absolute error (MAE), and adjusted R squared.

Evaluation Metrics	Water Temperature (°C)	Nitrate (μmol/L)	Phosphate (μmol/L)
RMSE	2.04	2.79	0.22
MAE	1.41	1.84	0.14
${R}_{adj}^{2}$	0.93	0.97	0.96

**Fig. 4:**
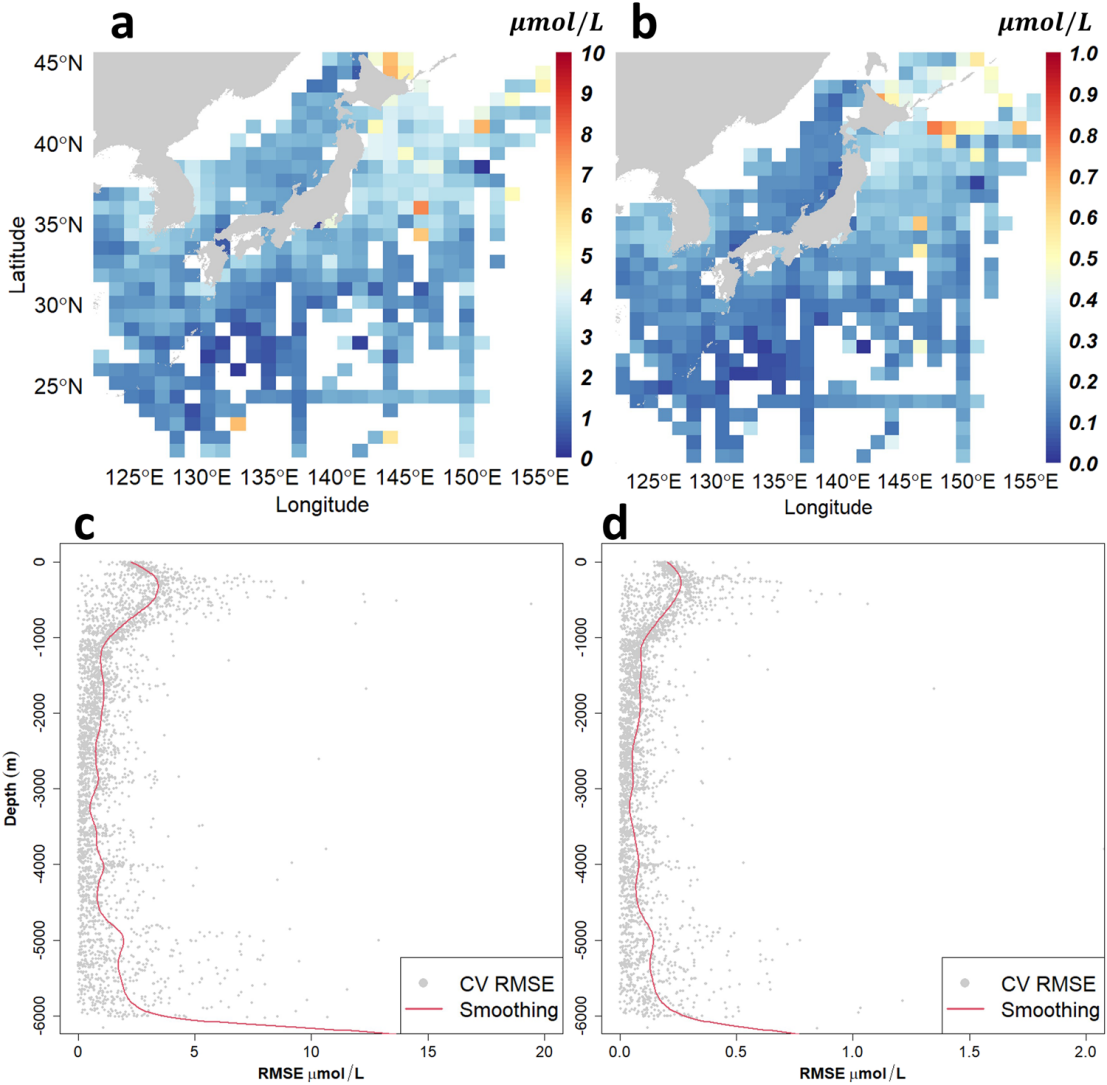
Error distribution for horizontal (**a,b**) and vertical (**c,d**) directions. a and c represent the error distribution of nitrate concentration, while b and d represent the error distribution of phosphate concentration. In areas with high data density, errors are relatively low. The vertical distribution of errors shows an increasing trend in the thermocline (approximately 300 m).

The performance of the model in predicting water temperature and nitrate and phosphate concentrations was evaluated using error metrics (RMSE, MAE, adjusted R-squared). Note that since various reanalysis datasets are available, sea water temperature estimates are not provided here, and only the performance evaluation results are presented as supporting information. The estimation errors for water temperature were 2.05 °C (RMSE), 1.42 °C (MAE), and 0.93 (${R}_{adj}^{2}$). The error metrics for nitrate concentration were 2.79 (RMSE), 1.84 (MAE), and 0.97 (${R}_{adj}^{2}$), and those for phosphate concentration were 0.22 μmol/L (RMSE), 0.14 μmol/L (MAE), and 0.96 (${R}_{adj}^{2}$). The spatial distribution of the errors showed that the area near Hokkaido in Japan had higher nutrient concentrations than other areas, with approximately 7 μmol/L for nitrate and approximately 0.8 μmol/L for phosphate(Fig. 4a,b).

The RMSE variation was analyzed according to depth(Fig. 4c,d). The RMSE was observed to be relatively higher within the 0–1000 m depth range, where the thermocline is located, but remained stable below a depth of 1000 m. However, an increase in RMSE was observed in the deep sea below 5000 m. The increased error in the thermocline and deep sea was attributed to the abrupt changes in nutrient concentration and lack of data, respectively.

**Fig. 5:**
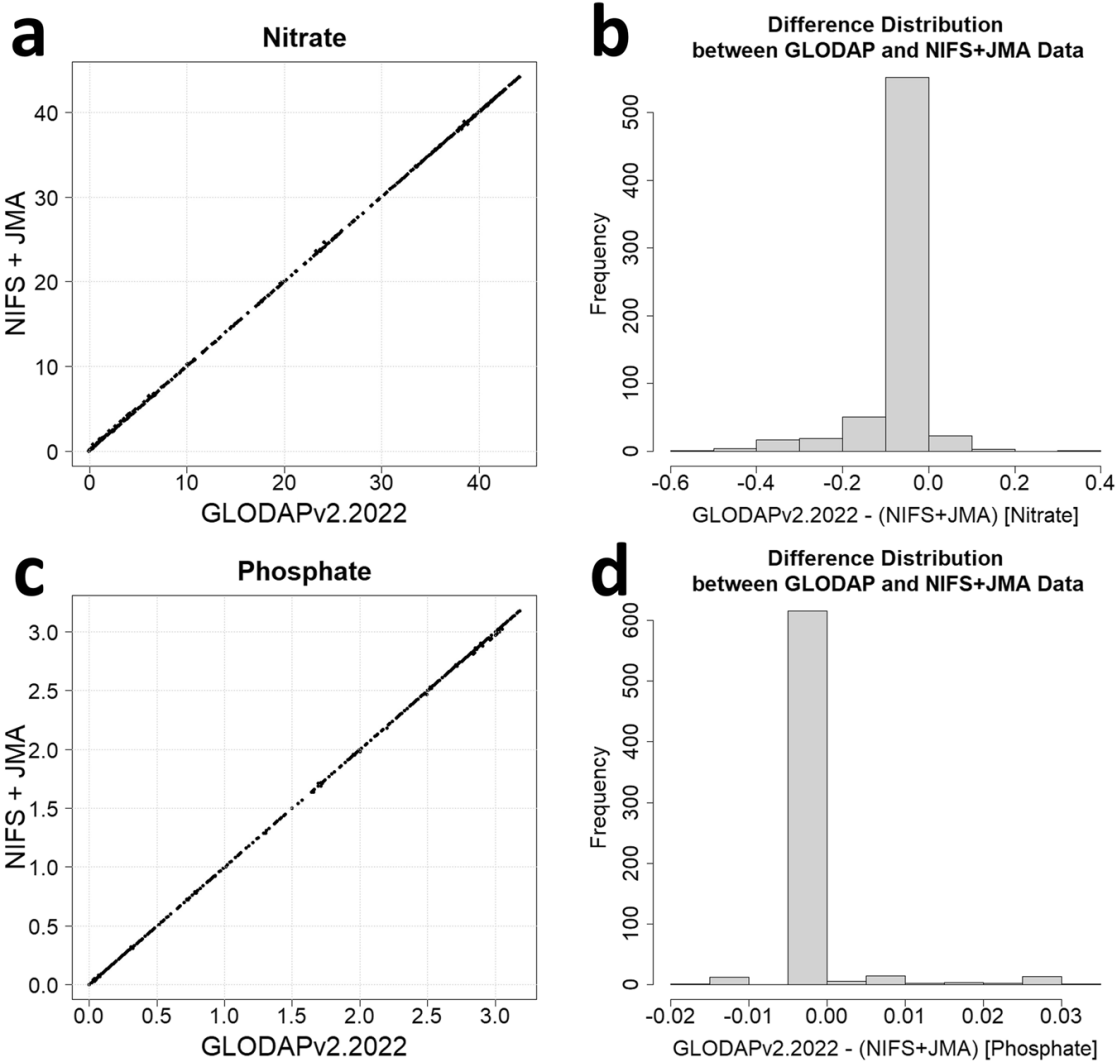
A comparison of nutrient data used for STK estimation (from NIFS and JMA) with spatiotemporally matched cruise data from GLODAPv2.2022. The results of the comparison for nitrate (**a,c**) and phosphate (**b,d**) are presented, with the distribution of differences converging towards a central value of 0.

Additionally, Compatibility with global-scale projects was assessed. The raw data utilized in this study was contrasted with the biogeochemical data product of GLODAPv2.2022(https://www.ncei.noaa.gov/data/oceans/ncei/ocads/data/0257247/)^59^. Since the data prior to 2010 was verified in previous research using CLIVAR and SIO datasets^11^, the focus was on data from 2010 onwards. A total of 671 data points with precisely matching longitude, latitude, depth and time were compared(Fig. 5).

**Fig. 6:**
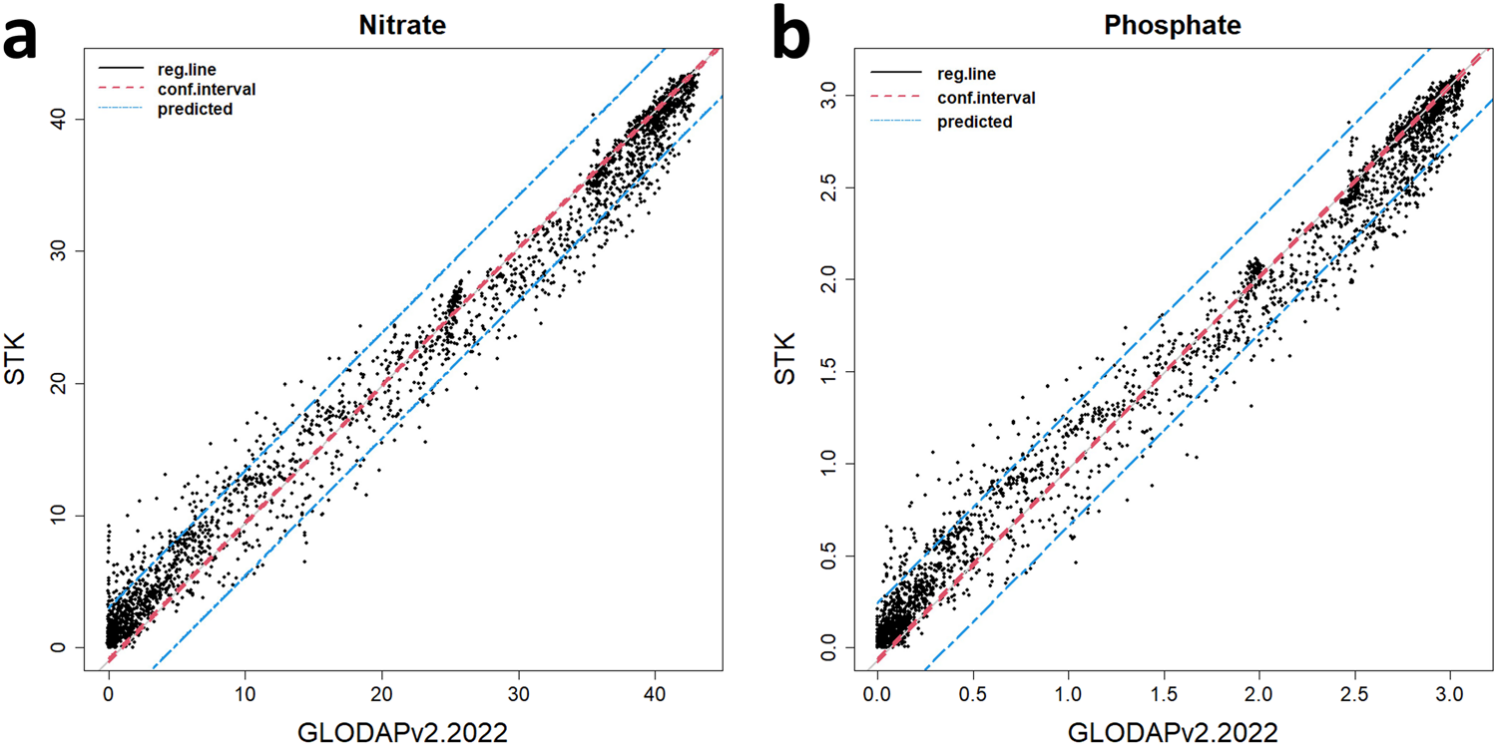
Results from a comparison of nitrate (**a**) and phosphate (**b**) gridded data estimated by STK from 2010 to 2019 with GLODAPv2.2022 data. Information from the linear model is represented by the linear regression line (black solid line), 95% confidence interval (red dotted line), and 95% prediction interval (blue dotted line).

Subsequently, the data estimated by STK was also compared with the GLODAPv2.2022 data(Fig. 6). Among the gridded data from the period 2010–2019, grids that were spatiotemporally closest to certain GLODAPv2.2022 data were compared. The grid data closest to the GLODAPv2.2022 data were identified, and those within the 5% quantile distance were selected. The criteria for selection were a horizontal distance of approximately 15 km, a vertical distance of about 16 m, and a time difference within roughly 9 days. For NO3, 2,652 data points were contrasted, yielding an ${R}_{adj}^{2}$ of 0.984 and a Residual Standard Error of around 2.03. For PO4, 2,676 data points were examined, with an ${R}_{adj}^{2}$ of 0.981 and a Residual Standard Error of approximately 0.158.

## Usage Notes

**Fig. 7:**
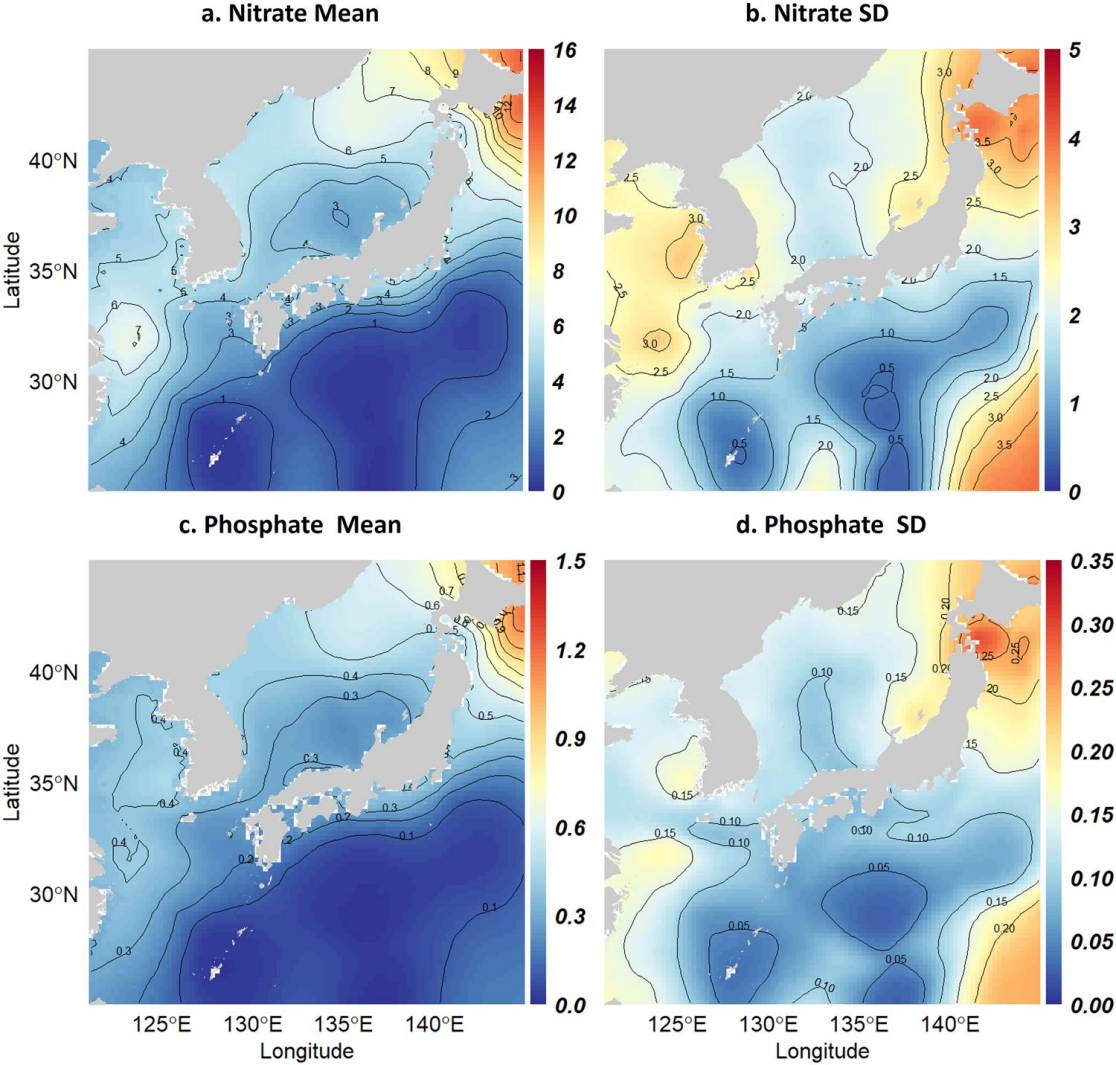
Spatial distribution of the mean and standard deviation of nitrate (**a,b**) and phosphate (**c,d**) concentrations during the target period of estimation (1980–2019). The mean concentration and variability are highest in the Yellow Sea and near Hokkaido, Japan. The high standard deviation observed in the northwest Pacific off the southeast coast of Japan is attributed to the lack of data.

**Fig. 8:**
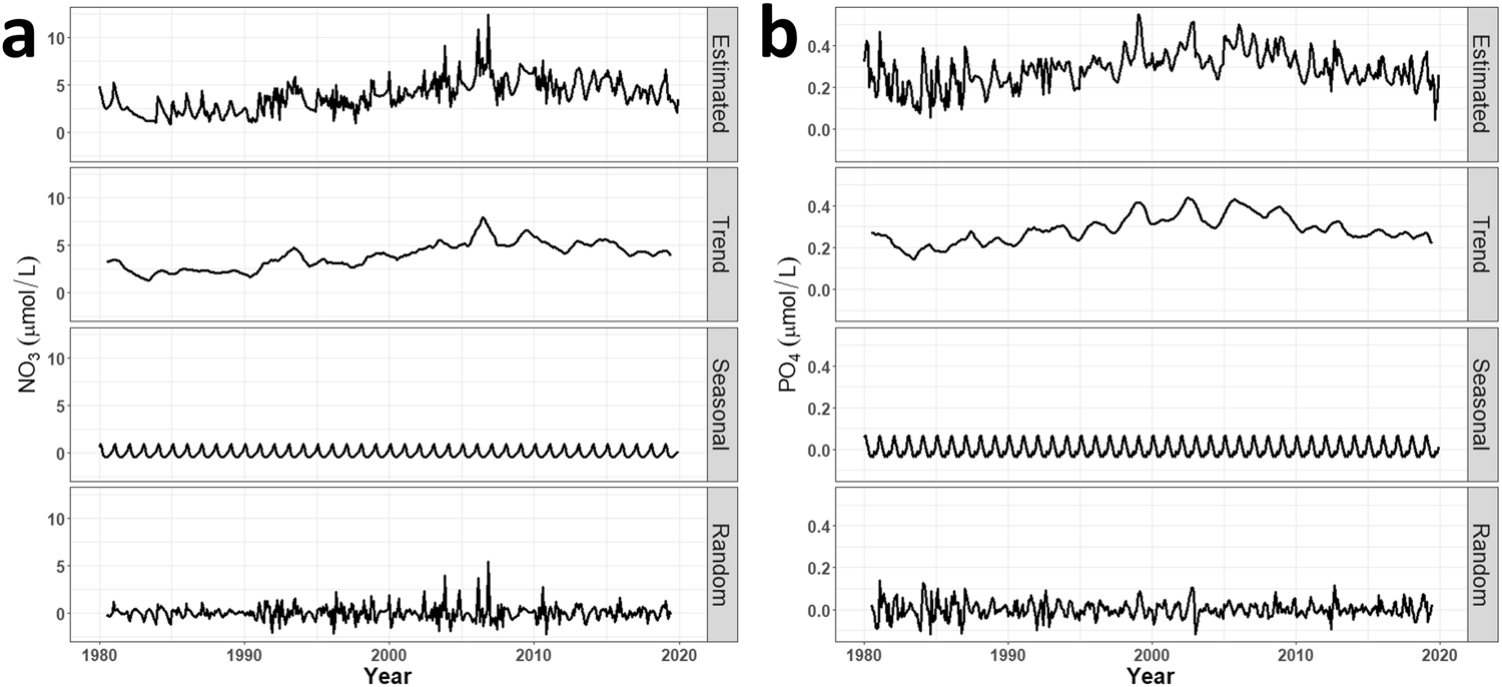
The time series decomposition of the estimated nitrate (**a**) and phosphate (**b**) concentrations in the Yellow Sea region is displayed, where they were decomposed into trend, seasonal, and residual components. These outcomes can be utilized for research on the sources of variability and trend analysis.

This dataset was used to assess the nutrient dynamics in select areas of the northwest Pacific, both locally and regionally (Fig. 7). Gridded data can be examined through basic statistical analysis and spatial statistical methods such as EOF. These data can also be utilized for comparison with biogeochemical modeling outcomes. The surface (0–50 m averaged) nitrate concentration trend estimated in this study corroborates the decreasing nitrate concentration trend observed in previous studies^20,21^ since approximately 2010 in the Yellow Sea (Fig. 8) .

**Table 4:** The system environment used for development and testing is presented.

	Originally Developed	Also Tested
**OS**	Windows 10 Enterprise	Linux Ubuntu 20.04
**System**	64 bit, x64 processor	
**CPU**	Intel i9-9900K 3.60 GHz 8 core 16 thread	Intel i9-10980XE 3.00 GHz 18 core 36 thread
**RAM**	64 GB	256 GB
**R version**	4.1.1	4.1.3

The main development system for the *STK* library utilized a Windows operating system and was also tested on a Linux (Ubuntu) system.

## Data Availability

The R code scripts and dataset are available on ‘Figshare’ for reproducibility^54^. The author’s GitHub online repository will be continuously updated to ensure sustainable usage of these codes(https://github.com/Gi-Seop/STK). **Tested system** All codes were tested in the following system environment (Table 4).
